# β-3AR W64R Polymorphism and 30-Minute Post-Challenge Plasma Glucose Levels in Obese Children

**DOI:** 10.4274/jcrpe.1629

**Published:** 2015-03-05

**Authors:** Hasibe Verdi, Sibel Tulgar Kınık, Yaprak Yılmaz Yalçın, Nursel Muratoğlu Şahin, Ayşe Canan Yazıcı, F. Belgin Ataç

**Affiliations:** 1 Başkent University Faculty of Medicine, Department of Medical Biology, Ankara, Turkey; 2 Başkent University Faculty of Medicine, Department of Pediatric Endocrinology, Ankara, Turkey; 3 Başkent University Faculty of Medicine, Department of Biostatistics, Ankara, Turkey

**Keywords:** obesity, children, β3-adrenergic receptor gene polymorphism, oral glucose tolerance test

## Abstract

**Objective:**

In this study, we aimed to investigate the association of W64R polymorphism of the β3-adrenergic receptor gene (β-3AR) with childhood obesity and related pathologies.

**Methods:**

β-3AR gene W64R genotyping was carried out in 251 children aged 6-18 years. Of these subjects, 130 were obese (62 boys) and 121 were normal-weight (53 boys). In the obese group, fasting lipids, glucose and insulin levels were measured. Oral glucose tolerance test (OGTT) was performed in 75 of the obese patients.

**Results:**

The frequency of W64R genotype was similar in obese and non-obese children. In obese children, relative body mass index, waist-to-hip ratio, serum lipid, glucose and insulin levels, as well as homeostasis model assessment of insulin resistance (HOMA-IR) scores were not different between Arg allele carriers (W64R and R64R) and noncarriers (W64W). In 75 obese children, OGTT results showed that Arg allele carriers had significantly higher 30-minute glucose levels (p=0.027).

**Conclusion:**

W64R polymorphism of the β-3AR gene is not associated with obesity and waist-to-hip ratio in Turkish children. Although there were no relationships between the genotypes and lipid, glucose/insulin levels or HOMA-IR, the presence of W64R variant seemed to have an unfavorable influence on early glucose excursion after glucose loading.

## INTRODUCTION

Obesity is a result of an imbalance between nutrient intake and energy expenditure. Increased positive energy balance causes fat storage. With increasing epidemics of obesity all over the world, recent research focused on both genetic and environmental factors affecting the energy balance in children and adolescents (1,2,3).

The sympathetic nervous system plays an important role in the regulation of energy expenditure. Catecholamines are powerful regulators of lipolysis and act via β-adrenoceptors (β-ARs). Thus, β-ARs play important roles in energy expenditure and body weight. Base variation in the β-3AR causes the substitution of the coding sequences from tryptophan (W) into arginine (R) in 64th position, a change that influences the affinity of the receptor to norepinephrine. Masuo et al (4) have reported close relationships between β-2 and β-3AR polymorphisms accompanying elevated sympathetic nervous activity, hypertension, obesity and insulin resistance in a longitudinal study. β-3ARs are located mainly in the adipose tissue and are involved in the regulation of lipolysis and thermogenesis by catecholamines, as well as in the development of obesity (5).

In a number of previous studies, β-3AR genotype (mainly W64R) was associated with obesity and related disorders such as hypertension, increased waist-to-hip ratio, cardiovascular disease, dyslipidemia, insulin resistance and metabolic syndrome in adults (6,7,8,9,10,11,12,13). However, these findings have not been confirmed in other studies (14,15,16).

In children, reported data are limited and discordant. In some studies with children, R64 allele was not found to be related to obesity (17,18,19). In contrast, in other studies, W64R variant was found to be associated with obesity (20,21,22).

In a recent study with obese children, W64R polymorphism was found to be significantly associated with metabolic syndrome components such as increased visceral fat, dyslipidemia, higher blood pressure (23). Although β-3AR genotype was found to be related with insulin resistance (IR), there is scarce data on results of oral glucose tolerance test (OGTT).

In this study, we aimed to investigate W64R polymorphism of the β-3AR gene in obese children and also the relationship between genotype and obesity-related metabolic disorders. The association between β-3AR genotype and glucose-insulin levels during OGTT was also investigated.

## METHODS

Two hundred fifty-one unrelated children and adolescents were enrolled in the study. Of these cases, 130 were obese and 121 constituted the healthy control group.

The DNA studies were conducted on peripheral blood for β-3AR W64R genotyping of the children in the obese and control groups. In the obese group, IR and dyslipidemia were investigated as keys to carbohydrate and lipid metabolism disorders.

All patients were clinically free of symptoms except for obesity and they were not taking any medication. Height was measured in all subjects using a standard wall-mounted stadiometer. Weight was measured with a calibrated electronic scale. Anthropometric data also included body mass index (BMI) estimation and waist and hip circumference measurements. BMI was calculated using the weight/height2 (kg/m2) formula. As defined by the National Center for Health Statistics (www.cdc.gov), children with a BMI value above the 95th percentile for age and sex were considered as obese. Relative BMI (relBMI) was calculated using the following formula: subject’s 

BMI x 100/50th percentile BMI for the subject’s age and sex. Children with a relBMI ≥120 were also accepted as obese (24) .

Glucose, lipid and insulin levels were assessed in venous blood following an overnight fast (10-12 h). Serum glucose levels were measured using the glucose hexokinase method. Serum low-density lipoprotein cholesterol, high-density lipoprotein cholesterol and triglyceride levels were studied using Roche diagnostics methods (GbmH, Germany). Serum insulin levels were measured using the chemiluminescence method (DPC, Los Angeles, CA, USA). The homeostasis model assessment of insulin resistance (HOMA-IR) score was calculated with the following formula: HOMA-IR=fasting serum insulin (µU/mL) x fasting plasma glucose (mmol/L)/22.5 (25).

A standard OGTT (1.75 g/kg or a maximum of 75 g of glucose) following a 3-day, high-carbohydrate diet (300 g/day) and a 12-hour overnight fast was performed in 75 obese children. For glucose and insulin assessments, blood samples were obtained at 0, 30, 60, 90 and 120 minutes after glucose administration. Plasma glucose levels were measured with the glucose oxidase method and a modified Trinder color reaction catalyzed by the peroxidase enzyme and insulin levels were measured with an immunoradiometric assay kit.

Glucose tolerance was classified as normal (fasting plasma glucose <100 mg/dL), prediabetes (fasting plasma glucose 100-126 mg/dL and/or impaired glucose tolerance 2-hour postload 140-200 mg/dL) and type 2 diabetes (fasting plasma glucose ≥126 mg/dL and/or 2-hour postload ≥200 mg/dL) (26).

The subjects were defined as IR based on insulin peak of ≥150 µU/mL and/or ≥75 µU/mL 2 hours after a glucose load and when the sum of insulin levels during the OGTT was higher than 300 µU/mL (27,28,29).

The study protocol was approved by the ethics committee of Başkent University and an informed consent from all participants was obtained.

Genotyping: Genomic DNA was prepared from leukocyte pellets by sodium dodecyl sulfate lysis, ammonium acetate extraction and ethanol precipitation. The primers used and the conditions for polymerase chain reaction (PCR) analysis were as described previously (30). The basepair (bp) PCR products were digested with MvaI. The uncut product (161 bp) showed the presence of the W allele. When the PCR product was cut into two fragments of 99 and 62 bp, the R allele was revealed.

### Data Analysis

Normality of distribution of the continuous variables was analyzed using the Shapiro-Wilk normality test. The Levene’s test was used to assess the homogeneity of variances in the different groups. If parametric test assumptions were available, two independent group means were compared by Student’s t-test. If these assumptions were not available, the Mann-Whitney U test was used for comparison of two group medians. The results were expressed as the number of observations (n) and the mean ± the standard deviation (X±Sx), median (M) and minimum-maximum values. Categorical variables were analyzed by Pearson χ2 test and Fisher’s exact test when determining the relationships between the variables. Data analyses were performed with SPSS software (Statistical Package for the Social Sciences, version 17.0, SSPS Inc, Chicago IL, USA). A p-value of <0.05 was considered statistically significant.

## RESULTS

The clinical characteristics of the study groups are given in Table 1. The screened β-3AR genotypes were not different in the obese and control groups and the frequencies of W/W, W/R, R/R genotypes were 88%, 9%, 3% in the obese group and 83%, 16%, 1% in the controls, respectively (p=0.142). The allele frequencies were also similar.

Genotypic distribution satisfied the Hardy-Weinberg equilibrium (χ2, p=0.9998) in the obese group. One hundred and eight (83.0%) subjects were classified as homozygous (W64/W64) and 22 (17.0%) individuals as R64-allele carriers (W64/R64 and R64/R64).

There were no relationships between the polymorphism genotypes and serum fasting glucose, insulin, lipid levels or HOMA scores in obese children (Table 2).

OGTT was performed in 75 obese children. In 4 patients, fasting glucose levels were between 101 and 106 mg/dL. In one patient, the 120-min glucose level was 206 mg/dL and in 11 patients, the 120 min glucose levels were between 140 and 200 mg/dL. 56 of 75 cases had IR. Polymorphism frequency was not different between children who had or did not have IR (p=0.5). Plasma glucose and insulin levels during OGTT were not different between R allele carriers and noncarriers except for 30-minute glucose levels. The mean of the serum glucose level at 30 minutes was significantly higher in the R allele carrier group (Table 3, p=0.027).

## DISCUSSION

Diverse results have been reported in studies on the relation between W64R polymorphism of the β-3AR gene and obesity performed in adults and children. In this study, we investigated the association between W64R polymorphism in the β-3ARgene and both obesity and obesity-related carbohydrate and lipid metabolism disorders in Turkish children. As a further step, we also investigated the relationships between W64R polymorphism of the β-3AR gene and glucose and insulin levels during OGTT in obese children.

The β-3AR gene is involved either directly or indirectly in lipid and glucose metabolism processes and may have an influence on endogenous energy balance and body mass regulation. β-3AR promotes lipolysis and thermogenesis by catecholamine release. Base variation in the β-3AR causes the substitution of the coding sequences from tryptophan into arginine in 64th position and thus influence the affinity of the receptor to norepinephrine. W64R variation of β-3AR is associated with lower metabolic resting rate, with abdominal obesity, weight gain and difficulty losing weight (4,5,8,31,32). However, these findings have not been confirmed in other studies (14,15,33). Interestingly, Genelhu et al (14) found that obese and hypertensive Brazilian adults with W64/W64 genotypes had elevated fasting plasma insulin levels and higher HOMA-IR scores. Such discordant results may be partially explained by ethnicity, age, or population differences in the studied samples. Højlund et al (16) studied W64R genotype of the β-3AR gene in male twins with a high similarity in genetic and environmental background. They found that the heterozygosity for the W64R variant is unlikely to increase the risk of obesity, insulin resistance or type 2 diabetes.

Studies in obese children are also discordant. In some studies with children, similar to our result, R64 allele was not found to be related with obesity (17,18,19). In contrast, some studies showed that the W64R variant was associated with obesity in children (20,21,22). In a recent study, W64R polymorphism was found to be significantly associated with increased visceral fat, dyslipidemia and higher blood pressure in obese children (34).

In the present study, we failed to show any relationship between β-3AR genotype and obesity in children. Also W64R polymorphism of the β-3AR gene was not found to be associated with obesity-related parameters such as insulin resistance, dyslipidemia and hepatosteatosis. This result needs to be interpreted with caution since the study groups are relatively small in number.

In our study, we have also investigated the association between W64R polymorphism and post-challenge glucose-insulin levels during OGTT. Our results showed that the 30-minute post-challenge glucose levels were significantly higher in obese children who were R allele carriers. Interestingly, fasting glucose, insulin and HOMA scores and other post-challenge glucose or insulin levels in obese children were not different from the controls, except for 30-minute glucose level.

Erhardt et al (34) performed OGTT in obese children but reported no differences in 30-, 60-, 90-, and 120-minute glucose or insulin levels with respect to β-3AR genotypes. Very similar results were also reported in a study in Polish children (35).

β-3AR genotype was found to be related with insulin resistance, but data on OGTT in these subjects are scarce. OGTT has also been often used to evaluate β-cell function and IR (36). Acute hyperglycemia in response to an oral glucose load has a suppressive effect on endothelium-dependent vasodilatation, leads to an increase in oxidative stress and in the magnitude of the inflammatory response in the vasculature, all of which are processes involved in atherogenesis. Choi et al (37) showed an association between post-challenge 30-minute glucose levels during OGTT and arterial stiffness in Korean adults in whom OGTT was performed to investigate fasting hyperglycemia. Urine albumin excretion is a marker for vascular damage. It has been shown that the 30-minute post-challenge plasma glucose level is associated with urine albumin excretion in males and in postmenopausal women with normal glucose regulation. Besides its effect on lipolysis and biological energy production, β-3AR may modulate peripheral vascular tone and increase the blood pressure (38). Some clinical studies pointed to a possible relationship between arterial hypertension and W64R polymorphism of the ADRB3 gene as well as to a relationship between this genotype and higher mortality among hypertensive patients (9,13). In another study, obesity and hypertension have been considered to be related to polymorphisms of the β-3AR gene (4). An important limitation of our study is that we have no data about the blood pressure levels of our patients.

In conclusion, our results showed that among obese children, R allele carriers have higher post-challenge 30-minute glucose levels. Our results warrant further support from studies on relationships between β-3AR polymorphism and acute glucose excursions and vascular tone impairment in obesity. Our findings may be a step in the clarification of the big puzzle of the molecular basis of obesity. Long-term follow-up of these children might help us to understand the interactions between polymorphism and metabolic disorders especially in view of glucose metabolism and also of hypertension. Further research is needed to identify W64R polymorphism as a new risk factor of childhood obesity and disorders related to glucose metabolism and/or cardiovascular disorders.

## Figures and Tables

**Table 1:**
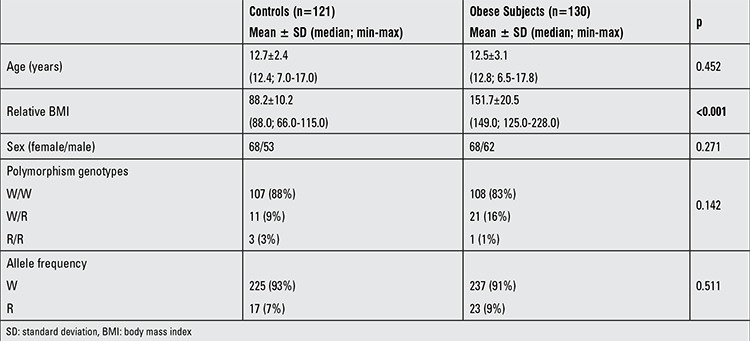
The clinical characteristics and genotype frequencies of the obese and control groups

**Table 2:**
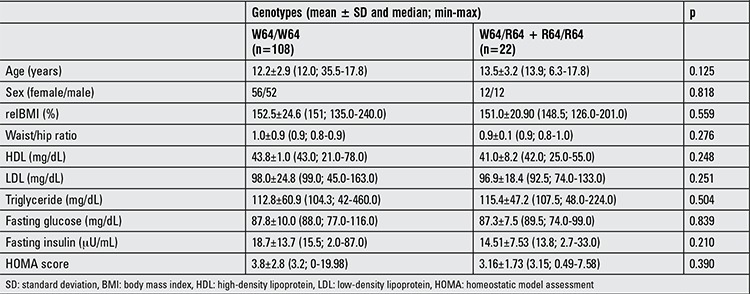
The clinical and laboratory characteristics of obese children with and without R allele

**Table 3:**
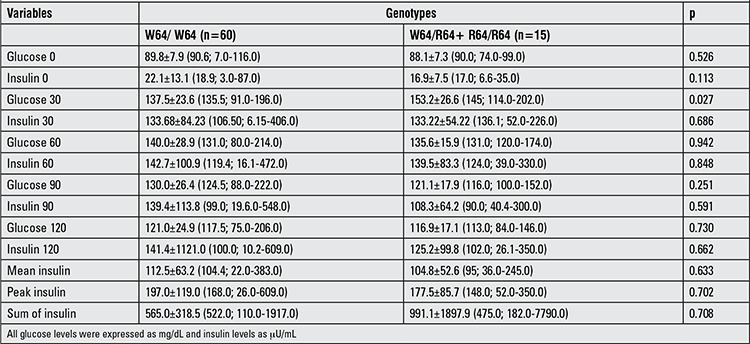
Glucose and lipid levels during oral glucose tolerance test (OGTT) in R allele carriers and non-carrier patients
